# Predicting the effect of CRISPR-Cas9-based epigenome editing

**DOI:** 10.1101/2023.10.03.560674

**Published:** 2023-10-03

**Authors:** Sanjit Singh Batra, Alan Cabrera, Jeffrey P. Spence, Isaac B. Hilton, Yun S. Song

**Affiliations:** 1 Computer Science Division, University of California, Berkeley; 2 Department of Bioengineering, Rice University; 3 Department of Genetics, Stanford University; 4 Department of BioSciences, Rice University; 5 Department of Statistics, University of California, Berkeley

## Abstract

Epigenetic regulation orchestrates mammalian transcription, but functional links between them remain elusive. To tackle this problem, we here use epigenomic and transcriptomic data from 13 ENCODE cell types to train machine learning models to predict gene expression from histone post-translational modifications (PTMs), achieving transcriptome-wide correlations of ~ 0.70 – 0.79 for most samples. In addition to recapitulating known associations between histone PTMs and expression patterns, our models predict that acetylation of histone subunit H3 lysine residue 27 (H3K27ac) near the transcription start site (TSS) significantly increases expression levels. To validate this prediction experimentally and investigate how engineered vs. natural deposition of H3K27ac might differentially affect expression, we apply the synthetic dCas9-p300 histone acetyltransferase system to 8 genes in the HEK293T cell line. Further, to facilitate model building, we perform MNase-seq to map genome-wide nucleosome occupancy levels in HEK293T. We observe that our models perform well in accurately ranking relative fold changes among genes in response to the dCas9-p300 system; however, their ability to rank fold changes within individual genes is noticeably diminished compared to predicting expression across cell types from their native epigenetic signatures. Our findings highlight the need for more comprehensive genome-scale epigenome editing datasets, better understanding of the actual modifications made by epigenome editing tools, and improved causal models that transfer better from endogenous cellular measurements to perturbation experiments. Together these improvements would facilitate the ability to understand and predictably control the dynamic human epigenome with consequences for human health.

## 1 Introduction

All cells within a multicellular organism have the same genetic sequence up to a minuscule number of somatic mutations. Yet, many cell types exist with diverse morphological and functional traits. Epigenetics is an important regulator and driver of this diversity by allowing differences in cellular state and gene expression despite having the same genotype (Taherian Fard and Ragan 2019). Indeed, cells traversing the trajectory from pluripotency through terminal differentiation have essentially the same genotype.

Epigenetic modifications such as post-translational modifications (PTMs) to histone core proteins are involved in many vital regulatory processes influencing genomic accessibility, nuclear compartmentalization and transcription factor binding and recognition (Reik et al. 2001; Kouzarides 2007; Gibney and Nolan 2010; Klemm et al. 2019; Hafner and Boettiger 2022; Zhang and Reinberg 2001). The Histone Code Hypothesis suggests that combinations of different histone PTMs specify distinct chromatin states thereby regulating gene expression (Strahl and Allis 2000; Jenuwein and Allis 2001).

The field of epigenome editing has produced new tools for understanding the outcomes of epigenetic perturbations that promise to be useful for therapeutics by enabling fine-tuned control of gene expression (Matharu and Ahituv 2020; Thakore et al. 2016; Goell and Hilton 2021; Stricker et al. 2017). Currently small molecule drugs are used to potently interfere with epigenetic regulation of gene expression. For example Vorinostat inhibits histone deacetylases thereby impacting the epigenetic landscape (Estey 2013; Yoon and Eom 2016). However, small molecules globally disrupt the epigenome and transcriptome, and therefore are not suitable for targeting individual dysregulated genes nor clarifying epigenetic regulatory mechanisms (Swaminathan et al. 2007). Meanwhile, numerous tools have been designed to harness catalytically dead Cas9 (dCas9) to target epigenetic modifiers to DNA sequences encoded in guide RNAs (gRNAs) (Jinek et al. 2012; Mali et al. 2013; Hilton et al. 2015; Stepper et al. 2017; Kwon et al. 2017; Li et al. 2021). CRISPR-Cas9-based epigenome editing strategies facilitate unprecedented, precise control of the epigenome and gene activation providing a path to epigenetic-based therapeutics (Cheng et al. 2019).

A major challenge for epigenome editing is designing gRNAs that can achieve a desired level of transcriptional or epigenetic modulation. Finding effective gRNAs currently typically requires expensive and low throughput experimental strategies (Mohr et al. 2016; Liu et al. 2020). An alternative approach would be to computationally model how epigenome editing impacts histone PTMs as well as how perturbing these PTMs would consequently impact gene expression.

To understand how histone PTMs relate to gene expression, large epigenetic and transcriptomic datasets are required. Advancements in high-throughput sequencing have allowed quantification of gene expression and profiling of histone PTMs. Large consortia have performed an extensive number of assays across a wide variety of cell types (ENCODE Project Consortium 2012; Roadmap Epigenomics Consortium et al. 2015; Barrett et al. 2012).

These include measurements of histone PTMs, transcription factor binding, gene expression, and chromatin accessibility. These data have enhanced our understanding of how histone PTMs and other chromatin dynamics impact transcriptional regulation (Keung et al. 2015; Rao et al. 2014; Holoch and Moazed 2015).

Studying the function of these histone PTMs, however, has been largely limited to statistical associations with gene expression, which may not capture causal relationships (Karlić et al. 2010; Stillman 2018; Singh et al. 2016). For example deep learning has been successful in predicting gene expression from epigenetic modifications, such as transcription factor binding (Schmidt et al. 2017), chromatin accessibility (Schmidt et al. 2020), histone PTMs (Singh et al. 2016; Sekhon et al. 2018; Frasca et al. 2022), and DNA methylation (Zhong et al. 2019). However, as statistical associations can be driven by non-causal mechanisms, it is unclear whether such computational models learn mechanistic, causal relationships between various epigenetic modifications and gene expression. Beyond modeling the relationship between histone PTMs and gene expression, to fully describe how a particular gRNA would affect gene expression, a model of how epigenome editing affects histone PTMs is also required. To our knowledge, there currently are no computational models that can accurately model, *in silico*, the impact of epigenome editing on histone PTMs.

**Figure 1:**
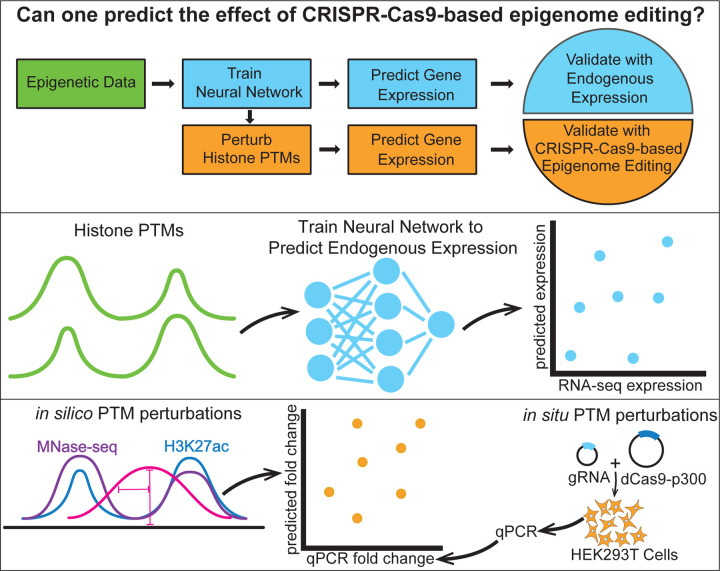
Schematic of the epigenome editing prediction pipeline. The pipeline uses epigenetic data to train models to predict endogenous gene expression. These models were used to predict fold change in gene expression based on perturbed histone PTM input data, and their predictions were validated using CRISPR-Cas9-based epigenome editing data.

Motivated by these observations, we explored models for how epigenome editing impacts histone PTMs as well as how histone PTMs impact gene expression. We used data available through ENCODE (Schreiber et al. 2020a; ENCODE Project Consortium 2012) to train a model of how histone PTMs impact gene expression. Our model is highly predictive of endogenous expression and learns an understanding of chromatin biology which is consistent with known patterns of various histone PTMs (Kimura 2013). To test this model in the context of epigenome editing, we generated perturbation data using the dCas9-p300 histone acetyltransferase system (Hilton et al. 2015). The dCas9-p300 system is thought to act primarily through local acetylation of histone lysine residues, particularly histone subunit H3 lysine residue 27 (H3K27ac). Therefore, we modeled the impact of dCas9-p300 on the epigenome as a local increase in the H3K27ac profile near the target site; since the precise effect of these perturbations is unknown, we tried a variety of potential modification patterns. We then applied our trained model to predict the impact of these putative H3K27ac modifications on gene expression (Fig. 1). We found that our models were effective in ranking relative fold changes among genes in response to the dCas9-p300 system, achieving a Spearman’s rank correlation of ~0.8. However, their performance in ranking fold changes within individual genes was less successful when compared to the prediction of gene expression across cell types from their native epigenetic signatures. We offer possible explanations in the discussion section.

## 2 Results

### 2.1 Histone PTM data are highly predictive of gene expression

Genome-scale datasets are required to train models to predict gene expression using histone PTMs. Therefore, we obtained histone PTM ChIP-seq and RNA-seq data for 13 different human cell types from ENCODE (Schreiber et al. 2020a; ENCODE Project Consortium 2012) (Supplementary Table S1). We inspected metagene plots (histone PTMs averaged across genes within gene expression quantiles) describing 6 histone PTMs in each of these 13 different cell types. Based on different overall signal levels across cell types, we concluded that batch effects, likely due to inconsistent sequencing depths, would need to be corrected prior to training models (Supplementary Fig. S1).

**Figure 2:**
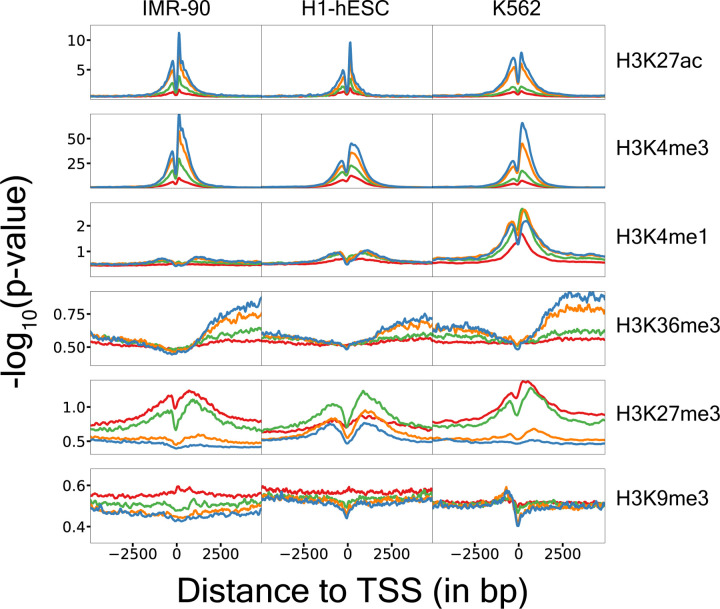
Metagene plots show histone PTMs are consistent across cell types and recapitulate established relationships between histone PTMs and gene expression. Colors represent genes binned into quantiles based on gene expression. Blue 75–100%, Orange 50–75%, Green 25–50%, Red 0–25% of gene expression within a cell type. The *y*-axis represents −*log*_10_(p-value) obtained from ChIP-seq data.

We corrected these batch effects by adapting S3norm (Xiang et al. 2020) (Section 4, Supplementary Fig. S2). These corrected histone PTM tracks were then used for the remainder of our analyses along with RNA-seq data for each of the 13 cell types (Section 4). Importantly, we observed that H3K27ac and H3K4me3 histone PTM signal strength positively covaried with gene expression quantile (representative cell types shown in Fig. 2; all cell types shown in Supplementary Fig. S3). Conversely, repressive histone PTMs such as H3K27me3 and H3K9me3 seemed strongly anti-correlated with gene expression quantile. Spatial patterns in the metagene plots for H3K36me3 suggested that this mark covaried more strongly with gene expression in the gene body than near the TSS. Taken together, these observations recapitulated the current understanding of these well-studied histone PTMs with respect to their associations to gene expression (Kimura 2013; Millán-Zambrano et al. 2022; Zhao et al. 2021).

### 2.2 Histone PTMs accurately predict endogenous gene expression

**Figure 3:**
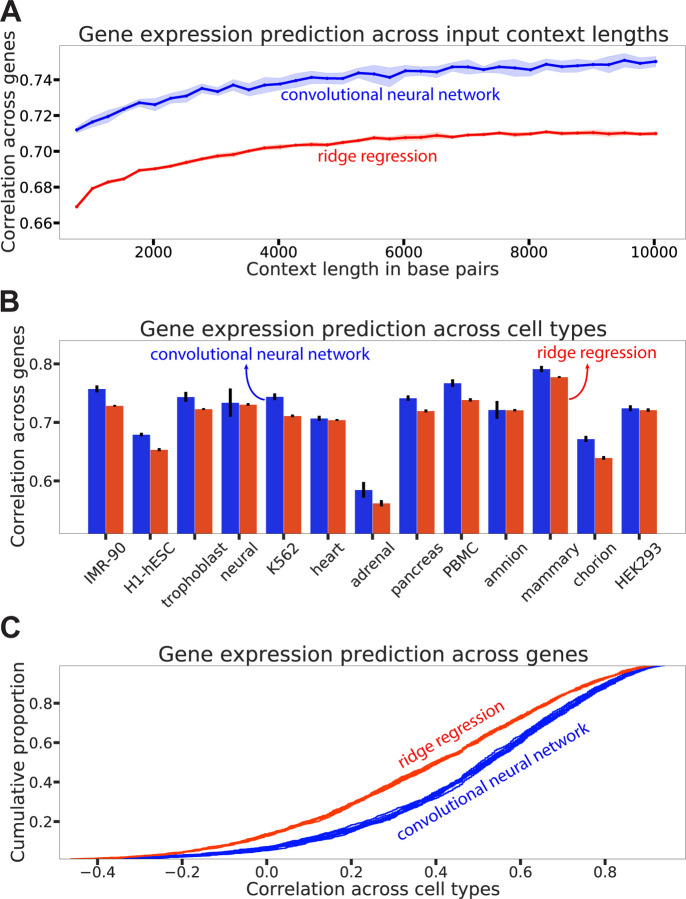
Histone PTMs accurately predict endogenous gene expression. **(A)** Spearman correlation on genes from held out chromosomes for different input context lengths, with all cell types pooled together. Blue curve is the mean across 10 computational replicates of CNNs and the red is the mean across 10 computational replicates of ridge regression. Shaded area represents standard deviation in the Spearman correlation across the 10 computational replicates. **(B)** Spearman correlation on genes of cell types held out during training. The bar plots represent the mean across 10 computational replicates and the error bars represent the corresponding standard deviations. **(C)** Distribution of Spearman correlations across genes, computed for each gene in test chromosomes by comparing predictions across the 13 cell types. The different curves represent 10 computational replicates for each model type.

To predict how epigenome editing affects gene expression, we first trained models to predict gene expression from endogenous histone PTMs. We trained several convolutional neural networks (CNNs) and ridge regression models to predict the gene expression of each gene in each of the 13 cell types, using only histone PTM data proximal to the TSS as features (Section 4, Fig. 2). We observed that Spearman’s rank correlation between the true gene expression and the models’ predicted gene expression on held-out chromosomes improves as the input context size increases; and for all input context sizes, the CNNs outperform ridge regression models (Fig. 3A). Therefore, for the remainder of the analyses, we use a context size of 10,000 base pairs.

To assess the models’ ability to generalize to unseen cell types, we trained a set of 10 models for each cell type. Concretely, we held out the histone PTMs for a given cell type during training and then tested the models on that held-out cell type.

We observed that the CNNs outperformed ridge regression models on this cross-cell type generalization task across essentially all cell types (Fig. 3B). The reduced performance on the adrenal cell type may be driven by a lower correlation of its epigenetic data with other cell types, particularly for H3K36me3 (Supplementary Fig. S5).

Although our models accurately predicted endogenous gene expression, this does not guarantee their ability to accurately predict the relationship between local histone PTM variations and with gene expression for a particular gene across different cell types. Therefore, we determined Spearman’s rank correlations between the observed expression and the predicted expression for each held-out gene across the different cell types. The distribution of these correlations suggests that overall the CNNs can better rank cell types by gene expression than ridge regression (Fig. 3C). In particular, the median cross-cell type correlation is ~0.53 for CNNs compared to ~0.39 for ridge regression.

### 2.3 Models recover established relationships between histone PTMs and gene expression

**Figure 4:**
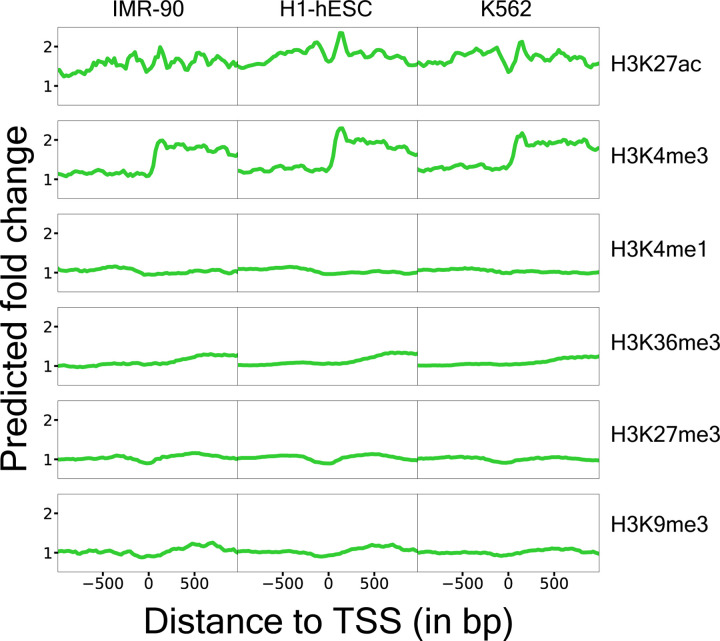
Features learned by gene expression models. Each point on the x-axis corresponds to *in silico* perturbation of that assay at that position and the y-axis measures the predicted fold change in gene expression, averaged across a set of 100 trained models. The fold changes were averaged across 500 randomly chosen genes.

We investigated what features of the data the models used to predict gene expression. For a given gene, we modified the input histone PTMs one-by-one at nucleosome-scale and measured the predicted fold change in gene expression (Fig. 4, Section 4).

We observed considerable changes to the predicted fold change upon modifying different histone PTMs. In particular, our CNN models predict that repressive marks such as H3K27me3 and H3K9me3 proximal to the TSS result in a slight decrease in expression. In contrast, activating histone PTMs such as H3K27ac and H3K4me3 result in an almost two-fold increase in predicted gene expression near the TSS. Additionally, we observed that H3K36me3 is predicted to increase expression, but only if it is deposited in the gene body, and the degree of activation gradually increases as it is deposited further inside of the gene body. The consistency of these observations with established mechanisms via which these histone PTMs modulate gene expression (Kimura 2013) lend credence to our gene expression models and show that these models learn the spatial patterns of histone PTMs.

### 2.4 dCas9-p300 differentially activates genes depending on gRNA-targeted site

**Figure 5:**
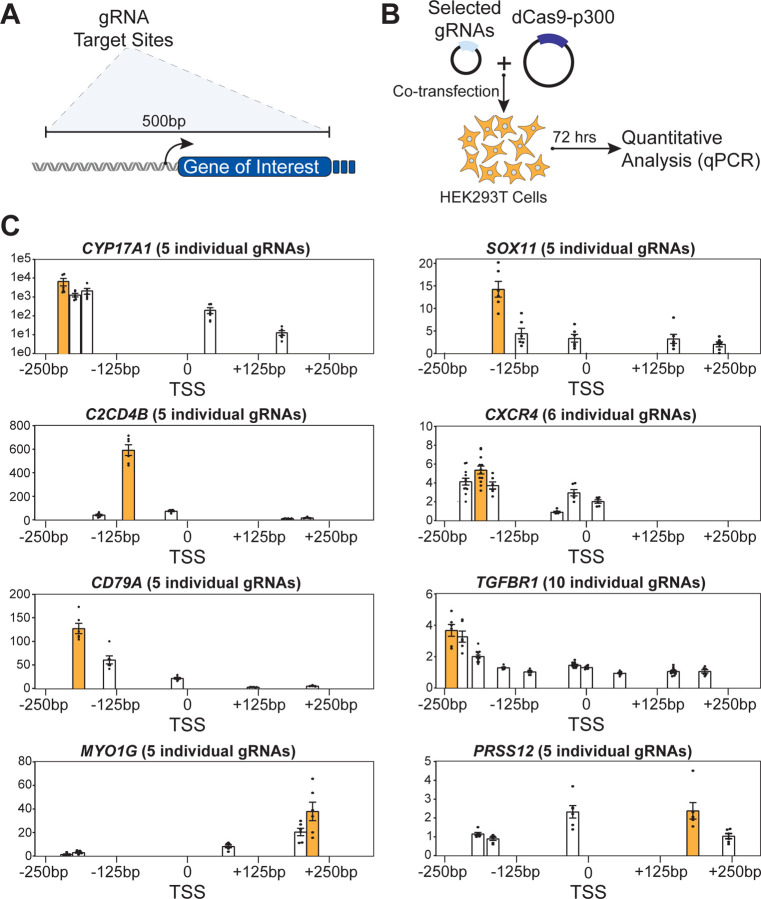
dCas9-p300 epigenome editing at eight endogenous genes identifies gene specific responses. The genes tested are CYP17A1, SOX11, C2CD4B, CXCR4, CD79A, TGFBR1, MYO1G, and PRSS12. (A) gRNA targeting +/− 250 bp of each gene were selected. (B) These Selected gRNA were individually co-transfected with dCas9-p300 with relative mRNA determined with qPCR. (C) Relative mRNA associated with selected guide position are displayed with the highest activating guide position marked in orange.

To test if our gene expression models could accurately predict the outcome of *in situ* epigenome editing experiments, we first generated dCas9-p300 data in the HEK293T cell line for 8 genes (Fig. 5). We assayed at least 5 gRNAs per gene with at least 3 replicates for each gRNA. We used the HEK293T cell line because it is a widely-used testbed for epigenome editing strategies (Hilton et al. 2015; Nuñez et al. 2021; O’Geen et al. 2017). Based on Fig. 2 and Fig. 4, the largest changes in H3K27ac across gene expression quantiles occur within 500 base pairs of the TSS, so we constrained gRNA targeting to this critical window. We filtered gRNAs for predicted specificity (Concordet and Haeussler 2018) and on-target activity scores (Sanson et al. 2018). Each gRNA was tested individually, and relative mRNA abundance was measured using quantitative PCR (qPCR).

We successfully increased gene expression of all 8 genes with fold change activation using the most effective respective gRNA for each gene ranging from 3-fold to ~6,500-fold relative to a non-targeting control gRNA (Fig. 5C). Some of this variation may be explained by differences in endogenous gene expression levels, with the targeting of lowly expressed genes resulting in higher fold change measurements (Supplementary Table S2), as observed previously (Wang et al. 2022a). Nevertheless, substantial variability was observed in gRNA efficacy for all targeted genes. In particular, two (MYO1G and PRSS12) out of eight genes had the most efficacious gRNA downstream of the TSS. This contrasts with other reports where targeting CRISPR/Cas based activators upstream of the TSS leads to the highest activation (Mohr et al. 2016; Gilbert et al. 2014).

These data indicate that the rules governing the outcomes for successful dCas9-p300-based epigenome editing – and subsequent increased transcriptional activation – are complex. For example, two gRNAs targeting within ~50 base pairs of each other on C2CD4B have a 100-fold difference in measured mRNA (Fig. 5C). Further, gRNAs targeting the same position in different genes can have vastly different effects. For instance, several gRNAs targeting ~250 base pairs upstream of the CYP17A1 TSS result in a high fold change while two gRNAs targeting roughly the same position in MYO1G failed to produce substantial activation (Fig. 5C).

### 2.5 Computationally predicting the outcome of dCas9-p300 epigenome editing experiments

To test the hypothesis that dCas9-p300 acts through the local deposition of H3K27ac, we modeled this process *in silico* and used these perturbations as inputs to our models trained on endogenous gene expression.

We modeled the effect of dCas9-p300 on histone PTMs based on evidence from the literature as well as additional experiments we performed. The key assumptions of this model are: 1) there exists steric hindrance of dCas9 by nucleosomes (Makasheva et al. 2021; Horlbeck et al. 2016; Isaac et al. 2016; Radzisheuskaya et al. 2016); 2) dCas9-p300 acts locally, altering H3K27ac levels near the gRNA target locus (Gemberling et al. 2021; Dominguez et al. 2022) (we adopted this simplifying assumption since off-target effects are unpredictable and unexplored); 3) dCas9-p300 can deposit H3K27ac at nucleosomes, as defined by MNase activity (see Section 4) (Segelle et al. 2022; Zhou et al. 2016). Our resulting *in silico* perturbation model had a number of free parameters that we briefly describe below. Wherever possible, we used values for these parameters obtained from the literature or tested a range of plausible values. For a more complete description of the model, see Section 4.

**Figure 6:**
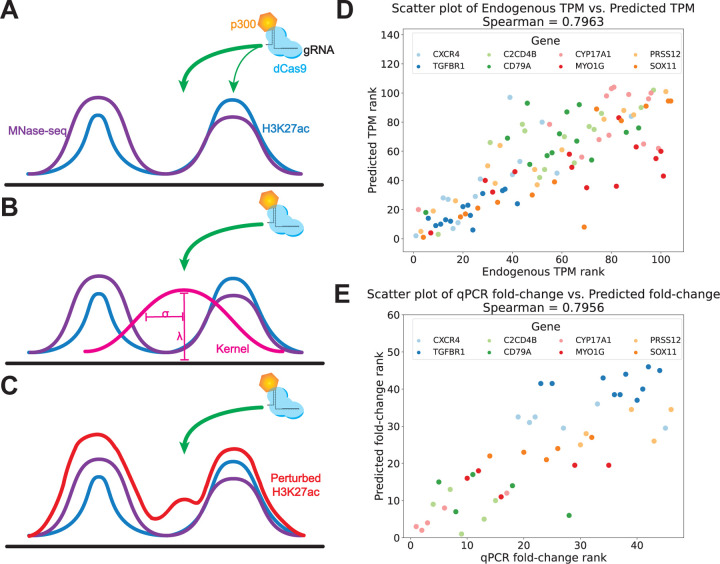
*In silico* model for dCas9-p300-based epigenome editing (A) dCas9-p300 is more likely to bind to a position not occupied by the nucleosome. Thicker green arrow represents higher probability of binding for a gRNA targeting that site. (B) The *in silico* perturbation is modeled as a Gaussian kernel parameterized by a standard deviation, *σ*, and the amount of H3K27ac deposited, *λ*. (C) The final perturbed H3K27ac is obtained by point-wise multiplication of the Gaussian kernel with nucleosome occupancy quantified by MNase activity since dCas9-p300 can only acetylate histones within nucleosomes. (D) Ranks for predicted and endogenous expression across 8 genes and 13 cell types. Higher ranks correspond to higher values. (E) Ranks for predicted and empirically measured expression fold changes following perturbation by dCas9-p300 for 8 genes in HEK293T cells. Higher ranks correspond to higher values.

The first component of our perturbation model is steric hindrance of dCas9-p300 by nucleosomes (Fig. 6A). Intuitively, if DNA is tightly wound around a nucleosome, the gRNA would be less likely to bind successfully. Mathematically, we modeled this as an inverse relationship between the amount of H3K27ac deposited and the MNase activity at the gRNA target locus.

It is widely assumed that dCas9-p300 activates genes through the local deposition of H3K27ac (Klann et al. 2017; Dominguez et al. 2022). To model this, we increased local levels of H3K27ac relative to endogenous levels according to a Gaussian kernel centered at the gRNA target locus (Fig. 6B). This adds acetylation primarily within a distance controlled by the standard deviation (*σ*) of the kernel. We performed CUT&RUN experiments (see Supplementary Material) that suggest that this distance is at least 1,000 base pairs (Supplementary Fig. S6). Since we also do not know the degree to which dCas9-p300 alters H3K27ac levels, we modeled this as another free parameter, *λ*, which we varied over a range of plausible values (Section 4).

Finally, we assumed that dCas9-p300 does not affect the positioning of nucleosomes and hence can only add H3K27ac at positions currently occupied by histones (Zhou et al. 2016). As such, we expect H3K27ac levels to only increase at loci where there is MNase activity. Concretely, we modulated the Gaussian kernel described above, by performing point-wise multiplication with MNase activity (Fig. 6C). Since nucleosome positioning plays a crucial role in our perturbation model, we generated, to our knowledge, the first MNase-seq data for the HEK293T cell line (see Supplementary Material).

To get a baseline of how well our perturbation model might be able to predict the effect of dCas9-p300 on gene expression, we considered the 13 distinct cell types as being analogous to natural perturbations of local histone PTMs. Across the 8 genes discussed above, which were excluded from the training set, we observed a Spearman’s rank correlation of ~0.8 between the endogenous expression and that predicted by our expression model (Fig. 6D). This correlation was in line with the correlation observed across the endogenous transcriptome (Fig. 3A,B). We further observed that our expression models were able to accurately rank gene expression across cell types within individual genes (Supplementary Fig. S7).

We then computed fold changes between the expression predicted using endogenous histone PTMs and the expression predicted using *in silico* perturbations of these histone PTMs. We observed that our models were effective in ranking relative fold changes across genes in response to dCas9-p300, achieving a Spearman’s rank correlation of ~0.8 between these predicted fold changes and the experimentally determined mRNA fold changes induced by dCas9-p300 (Fig. 6E). However, the performance in ranking fold changes within individual genes was less accurate (Supplementary Fig. S8) when compared to the prediction of cell-type-specific gene expression from native epigenetic signatures (Supplementary Fig. S7).

## 3 Discussion

Here, we sought to investigate whether we could predict how targeted epigenome editing affects endogenous gene expression. First, we collected data from ENCODE which reflects how post-translational modifications (PTMs) to histones covary with gene expression across cell types. We trained models to predict endogenous gene expression from these histone PTMs and found that these models were highly predictive (Fig. 3). We further showed that such models learned known relationships between histone PTMs and gene expression (Fig. 4). To test whether these expression models could predict the outcomes of epigenome editing experiments, we generated dCas9-p300 epigenome editing data in the HEK293T cell line for eight genes along with genome-wide MNase-seq data for this testbed cell line. We anticipate that the genome-wide nucleosome occupancy information for the HEK293T cell line provided by our MNase-seq experiment will be a useful resource for the genomics community.

We modeled dCas9-p300’s impact on local H3K27ac using a variety of parameter choices and found that these models accurately predicted fold changes across genes. However, they were less accurate at predicting the outcome of these experiments within a given gene, as compared to predicting gene expression from the endogenous epigenetic signatures (Supplementary Fig. S8, Supplementary Fig. S7). Since the endogenous epigenetic signatures could be different across genes, these *global* factors might drive the models’ accurate inter-gene fold change prediction accuracy. However, since ranking fold changes within a gene requires a detailed understanding of the epigenetic profiles before and after dCas9-p300 epigenome editing, the reduction in performance from predicting endogenous expression to predicting the outcome of epigenome editing experiments is likely explained by one or more of the following hypotheses: 1) dCas9-p300 activates gene expression by mechanisms other than the *local* acetylation of H3K27 or dCas9-p300 functions differently from native p300; 2) differences in gRNA efficacy are not accurately explained by existing computational scores; or 3) our models, trained on endogenous gene expression across various cell types, failed to generalize even if dCas9-p300 perturbations are correctly modeled. We discuss these possible explanations more in depth below.

We considered numerous models of how dCas9-p300 affects local histone PTMs. These models span current hypotheses of how dCas9-p300 alters local histone PTMs such as H3K27ac. The poor generalization of our models in predicting intra-gene epigenome editing fold changes could be explained by dCas9-p300 acting via mechanisms beyond local acetylation of histone proteins and H3K27 (Zhao et al. 2021). For example, p300 is a promiscuous lysine acetyltransferase and dCas9-p300 could be broadly acetylating across the proteome impacting *trans* factors (Weinert et al. 2018). Alternatively, local acetylation could be contingent on unmodeled factors such as *trans*-acting proteins or other histone PTMs present at the locus (Zhao et al. 2021; Zheng et al. 2021). Furthermore, the genome-wide specificity of dCas9-p300-mediated histone acetylation – although likely better than small molecule-based perturbations – remains imperfect (Gemberling et al. 2021; Dominguez et al. 2022). Our inability to accurately predict the relative fold change of different gRNAs targeting the same gene suggests that these unmodeled factors would have to differentially affect neighboring loci within the same gene. This highlights that the current understanding of the mechanism via which dCas9-p300 drives gene expression is potentially incomplete. To better understand this mechanism, it would be immensely helpful to generate a compendium of histone PTM profiles before and after performing epigenome editing, which would enable us to train better machine learning models to predict the impact of dCas9-p300 on gene expression.

Another possible explanation for the drop in accuracy is varying gRNA efficacies. For example, gRNAs might have different levels of on-target and off-target effects. Although we ensured that all of the gRNAs used in generating the dCas9-p300 epigenome editing data were predicted to have high on-target and low off-target scores, we observed examples of gRNAs that targeted roughly the same genomic position but had vastly different impacts on gene expression. This suggests that these differences could be driven by inconsistencies in gRNA efficacy instead of local acetylation dynamics. Generating a large number of pairs of gRNAs, such as through CRISPR screens (Schmidt et al. 2022), targeting nearby positions could help to elucidate the factors that drive differential gRNA efficacy for epigenome editing.

The ambiguity in how to accurately model the impact of epigenome editing stands in contrast to the simpler case of DNA sequence changes, where perturbations are relatively trivial to model. Indeed, dCas9-p300 changes histone PTMs in complex ways rendering the modeling of such perturbations much more challenging. In contrast, models like Enformer (Avsec et al. 2021) that predict gene expression directly from DNA sequence may be able to generalize to DNA sequence perturbations better due to their relative simplicity.

Lastly, it is possible that our models of how histone PTMs affect gene expression may not generalize to the types of histone PTM profiles generated by epigenome editing experiments. One source of such a generalization error is failing to learn a *causal* model. Since we trained our models on endogenous gene expression across multiple cell types, our models may be inferring non-causal relationships. For example, epigenetic regulators within a cell could deposit histone PTMs in response to high gene expression(Wang et al. 2022b; Williams and Gehring 2020; Mazzone et al. 2019). In this case, our models would learn that this histone PTM should lead to a higher gene expression but in reality the causality is reversed. Another source of generalization error could be extrapolating beyond the range of the training data. Massively increasing the amount of H3K27ac at a locus may make a gene look different than any other endogenous gene observed during training. Regression approaches including neural networks are known to have limitations in extrapolation (Xu et al. 2020).

Our research indicates that we can predict endogenous gene expression accurately based on histone PTMs. By creating a comprehensive dataset of epigenome editing, which assays histone PTMs before and after *in situ* perturbations, we can enhance machine learning models. This will improve our understanding of the effects of dCas9-p300 on gene expression and assist in the design of gRNAs for achieving fine-tuned control over gene expression levels. These advancements are vital for devising experiments that deepen our mechanistic insight and offer effective strategies for human epigenome editing.

## 4 Materials and Methods

### 4.1 Data preparation

We obtained − log_10_(p-value) ChIP-seq tracks created by running the MACS2 peak-caller (Feng et al. 2012) on read count data, from the ENCODE Imputation Challenge (Schreiber et al. 2020a). For three tracks where data were not available, we downloaded Avocado (Schreiber et al. 2020b) imputations from the ENCODE data portal (ENCODE Project Consortium 2012). We binned each epigenetic track at 25 base pair resolution and pre-processed them with an additional log operation before inputting them into the models for training. We downloaded polyA-plus RNA-seq gene expression Transcripts Per Million (TPM) values for each of the 13 cell types in Table S1, from the ENCODE data portal (ENCODE Project Consortium 2012) and preprocessed them with a log operation.

### 4.2 Normalizing *p*-values by adapting S3norm

We assigned IMR-90 to be a reference cell type, for each of the 6 histone PTMs and kept its p-values unchanged. We then performed a transformation for each of the remaining cell types adapted from the core technique developed by S3norm (Xiang et al. 2020), in order to normalize each histone PTM track in each of these remaining cell types, with respect to the corresponding histone PTM track in IMR-90.

First, we computed *peaks* in both, the reference as well as the target cell type. *Peaks* were defined as the 25 base pair bins corresponding to FDR-adjusted p-values less than 0.05 (Benjamini and Hochberg 1995). For histone PTM tracks that were obtained from Avocado imputations (due to lack of availability of experimental data), *peaks* were defined to be the 1000 bins containing the smallest Avocado imputed p-values (based on suggestions from the authors of Avocado (Schreiber et al. 2020b)). All the remaining bins were defined to be *background*, for both, the reference as well as the target cell types. We then computed the list of *peaks* that were common to both the reference and the target cell types. These were termed, *common peaks*. Similarly, we defined *common background* as the list of bins that were assigned to be *background* in both, the reference as well as the target cell types.

The S3norm method was designed to work with count data, which is always ≥ 1. However, the tone PTM tracks, which are represented as − log_10_(*p*-values), are not guaranteed to always be ≥ 1, hence, we transformed all the histone PTM tracks by adding 1 to the − log_10_(*p*-values), in both the reference as well as the target cell types.

Additionally, since the histone PTM tracks obtained from imputations performed by Avocado were not guaranteed to be distributed similar to experimental − log_10_(*p*-values), we scaled all the histone PTM tracks (both experimental as well as Avocado imputations) by dividing them by the minimum observed value in *common peaks* and *common background*, in order to bring experimental data and Avocado imputations onto a similar footing. In particular, before applying the S3norm normalization, we transformed − log_10_(p-values) in *common peaks* and *common background* for both the reference as well as the target cell type as following:

(1) $$\text{TransformedCommonPeak}{\text{s}}_{i,\text{reference}}=\frac{1+\text{CommonPeak}{\text{s}}_{i,\text{reference}}}{\min_{i}\left(\text{CommonPeak}{\text{s}}_{i,\text{reference}}\right)}$$

(2) $$\text{TransformedCommonPeak}{\text{s}}_{i,\text{target}}=\frac{1+\text{CommonPeak}{\text{s}}_{i,\text{target}}}{\min_{i}\left(\text{CommonPeak}{\text{s}}_{i,\text{target}}\right)}$$

(3) $$\text{TransformedCommonBackgroun}{\text{d}}_{i,\text{reference}}=\max\left(\frac{1+\text{CommonBackgroun}{\text{d}}_{i,\text{reference}}}{\min_{i}\left(\text{CommonBackgroun}{\text{d}}_{i,\text{reference}}\right)},0\right)$$

(4) $$\text{TransformedCommonBackgroun}{\text{d}}_{i,\text{target}}=\max\left(\frac{1+\text{CommonBackgroun}{\text{d}}_{i,\text{target}}}{\min_{i}\left(\text{CommonBackgroun}{\text{d}}_{i,\text{target}}\right)},0\right)$$

The normalization procedure of S3norm then wishes to find two positive parameters, *α* and *β* that are to be learned from the data such that both the following equations are satisfied:

(5) $$\text{mean}\left(\text{TransformedCommonPeak}{\text{s}}_{\text{reference}}\right)=\text{mean}\left(\alpha \times \text{TransformedCommonPeak}{{\text{s}}^{\beta }}_{\text{target}}\right)$$

(6) $$\text{mean}\left(\text{TransformedCommonBackgroun}{\text{d}}_{\text{reference}}\right)=\text{mean}\left(\alpha \times \text{TransformedCommonBackgroun}{\text{d}}_{\text{target}}^{\beta }\right)$$

Specifically, *α* is a scale factor that shifts the transformed − log_10_(*p*-values) of the target data set in log scale, and *β* is a power transformation parameter that rotates the transformed − log_10_(*p*-values) of the target data set in log scale (Supplementary Fig. S2). There is one and only one set of values for *α* and *β* that can simultaneously satisfy both the above equations for *common peaks* and the *common background* (Xiang et al. 2020).

The values of *α* and *β* were estimated by the Powell minimization method implemented in scipy (Fletcher and Powell 1963; Virtanen et al. 2020). The resulting normalized − log_10_(*p*-values) were used for all downstream analyses (Supplementary Fig. S3).

### 4.3 Training endogenous gene expression models

We trained convolutional neural network and ridge regression models, each, to predict gene expression using histone PTM tracks. Input features for each gene were centered at its TSS. We used an input context size of 10, 000 base pairs for all analyses subsequent to Fig. 3. For all analyses we obtained predictions from our models by averaging predictions ensembled across 100 computational replicates.

To train convolutional neural network models, the normalized histone PTM tracks for each gene were processed with successive convolutional blocks. Each convolutional block consisted of a batch-normalization layer, rectified linear units (ReLU), a convolutional layer consisting of 32 convolutional kernels, each of width 5, followed by a dropout with 0.1 probability. Finally a pooling layer was applied to gradually reduce the dimension of the features. After being processed with 5 such convolutional blocks, the output was flattened and passed through a fully connected layer consisting of 16 neurons and a ReLU activation. This was ultimately processed with a fully connected layer with a single output and a linear activation (since this was a regression task). The models were trained with a mean squared error loss using the Adam optimizer with a learning rate of 0.001 for the first 50 epochs and 0.0005 for the remaining 50 epochs. Training convolutional neural network models took about 1.5 hours on 1 NVIDIA A100 Tensor Core GPU.

### 4.4 Interrogating the features learned by CNNs

To see how different features affected predicted levels of expression, we systematically perturbed each input feature and determined how much the perturbation affected predicted expression levels. To be concrete, we denoted the epigenetic feature at position *i* of gene *g* in cell type *CT* as $E_{i}^{CT,g}$. We then defined a perturbation function that added a scalar value of *λ*_0_ = 2500 to the epigenetic features within 3 bins of a focal position, say, *j*:

$$F_{j}\left(E_{1}^{CT,g},\ldots ,E_{W}^{CT,g}\right):=\left(E_{1}^{CT,g},\ldots ,E_{j-3}^{CT,g}+\lambda_{0},\ldots ,E_{j+3}^{CT,g}+\lambda_{0},\ldots ,E_{W}^{CT,g}\right),$$

recalling that *W* is the number of bins of 25 base pairs considered by our models, which is set to 401, corresponding to a 10,000 base pair input context length, for all analyses subsequent to Fig. 3. These perturbations corresponded to ~ 150 base pairs which is roughly the length of DNA wrapped around a nucleosome.

To produce Fig. 4, we applied the above perturbation functions, $F_{1},\ldots ,F_{W}$ to a histone PTM track of interest, and the measured the fold change in predicted expression. To account for differences in the endogenous histone PTM tracks between genes, we averaged these fold changes across 500 randomly chosen genes.

### 4.5 *In silico* modeling of dCas9-p300-based epigenome editing

Our model of how dCas9-p300 perturbs local histone PTMs has three separate components. We describe each of these components in turn, and then present the full model below. Throughout, we write *j* for the position that the gRNA targets.

First, we modeled steric hindrance of dCas9 due to nucleosomes. We used MNase-seq signal strength as a proxy for nucleosome occupancy. Letting m_*j*_ be the MNase-seq read coverage at the gRNA binding site, we modeled steric hindrance by scaling the acetylation activity of dCas9-p300 by a factor of exp (−5 × *m*_*j*_).

Second, we assumed that dCas9-p300 primarily alters the levels of H3K27ac only locally. As such, we modeled the acetylation activity of dCas9-p300 at a particular locus as a Gaussian kernel centered at the gRNA. Concretely, the acetylation activity at position *i* is multiplied by a factor of $\exp\left(-{\left(i-j\right)}^{2}/2\sigma^{2}\right)$, where *σ*^2^ is a parameter of the model.

Finally, we assumed that dCas9-p300 can only acetylate histones where they currently are – it cannot move histones or increase H3K27ac levels outside of histones. To model this mathematically, we multiplied the acetylation activity at site *i* by the MNase read coverage, *m*_*i*_. Therefore, if the MNase read coverage is 0 (i.e., there is no evidence of histones at that locus) then the amount of H3K27ac added to that position is also 0.

Putting this all together, for a guide targeting at position *j*, the effect on H3K27ac levels at position *i* is proportional to

$$\exp\left(-5m_{j}\right)\times \exp\left[\frac{-{\left(i-j\right)}^{2}}{2\sigma^{2}}\right]\times m_{i}$$

The constant of proportionality (i.e., how strong we expect dCas9-p300 to be overall) is treated as another free parameter, which we denote by *λ*.

ENCODE has epigenetic data for the HEK293 cell line, but we performed our dCas9-p300 perturbations in the HEK293T cell line. As such, we used the HEK293 histone PTM as well as RNA-seq data as a stand in for the HEK293T histone PTM and RNA-seq levels. This substitution is justified as gene expression levels for HEK293 and HEK293T are highly concordant (Supplementary Table S2). Indeed the Spearman’s rank correlation between expression levels for HEK293 and two independent measurements of expression levels in HEK293T are 0.86 and 0.88, which are comparable to the correlation between the two independent experiments in HEK293T (*ρ* = 0.92). That is, the correlation across experiments within HEK293T cells is only slightly higher than the correlation between HEK293 and and HEK293T, suggesting that cross-cell type differences between HEK293 and HEK293T are on the same order as the inherent experimental and biological noise within a single cell type.

### 4.6 Experimental procedure

The details of dCas9-p300 epigenome editing, qPCR, CUT&RUN, and MNase-seq experiments are provided in the Supplementary Material.
